# Late Tricuspid Regurgitation after Percutaneous Transcatheter Closure of Ventricular Septal Defect: an Educational Presentation

**DOI:** 10.21470/1678-9741-2020-0172

**Published:** 2021

**Authors:** Kamil Sarkislali, Afksendyios Kalangos

**Affiliations:** 1 Department of Cardiac and Vascular Surgery, Istanbul Education Research Hospital, Istanbul, Turkey.; 2 Department of Cardiac and Vascular Surgery, Koc University Medical Faculty, Istanbul, Turkey.

**Keywords:** Heart Septal Defects, Ventricular, Echocardiography, Heart Valve Diseases, Early Diagnosis, Tricuspid Valve Insufficiency

## Abstract

Transcatheter closure of ventricular septal defects (VSD) is not out of complications. Late complications are rare, but important, and sometimes require surgical correction. Herein, we report a case of tricuspid regurgitation as a complication of transcatheter VSD closure. The patient underwent successful surgery. Postoperative course was satisfactory. Echocardiographic examination revealed well-functioning tricuspid valve. We present this case since valve regurgitation after transcatheter procedure requiring surgery is an uncommon but significant complication due to heart failure risk. Even in the absence of any clinical finding, post-procedural close follow-up is important for early diagnosis of the problem to prevent the aforementioned risk.

**Table:** 

Abbreviations, acronyms & symbols	
**LV**	**= Left ventricle**
**RA**	**= Right atrium**
**RV**	**= Right ventricle**
**VSD**	**= Ventricular septal defects**

## INTRODUCTION

Transcatheter closure of ventricular septal defects (VSD) is safe but not out of complications. Atrioventricular block and new-onset valve regurgitation are major post-procedural problems. Tricuspid regurgitation requiring surgery is an extremely rare iatrogenic complication^[1]^. The degree of valve insufficiency may vary according to the severity of the iatrogenic event. This complication may result in heart failure, which may be a life-threatening event. Management of the condition before development of heart failure is easier and more cost effective than management of heart failure and it presents good results with better quality of life. Herein, we present a case of a patient who underwent successful surgery before development of heart failure.

**Fig. 1:**
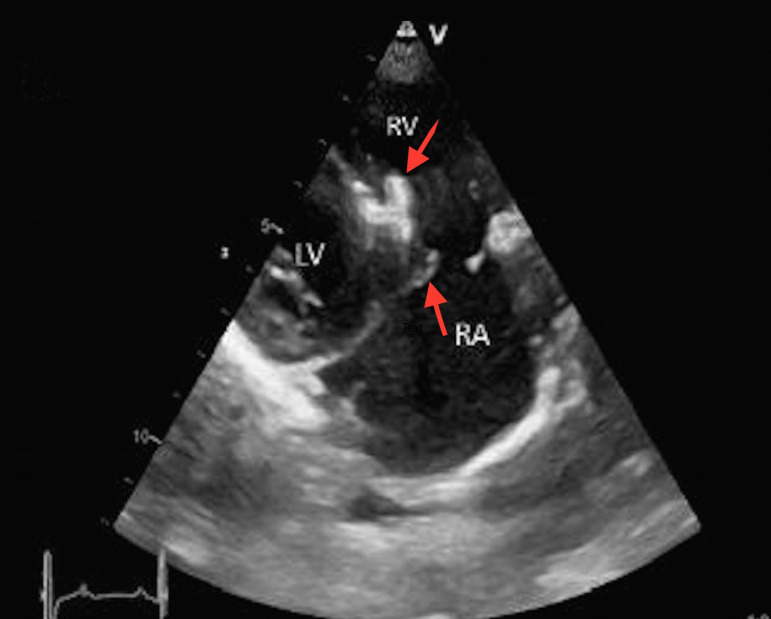
Transthoracic two-dimensional echocardiographic image of occluder device (upper arrow) and septal leaflet (lower arrow) entrapment during systole. LV=left ventricle; RA=right atrium; RV=right ventricle

A 10-year-old girl was admitted to our center with complaints of palpitation and shortness of breath. Medical history revealed transcatheter closure of VSD, three years ago. Physical examination showed tachycardia and 2-3/4 systolic regurgitant murmur along the left and right lower sternal borders. Cardiac rhythm was normal sinus rhythm and PR interval was within normal range. Liver was slightly palpable on the right subcostal region showing mild hepatomegaly. After careful medical evaluation, tricuspid regurgitation and probable residual VSD were diagnosed. Echocardiography showed coaptation failure of tricuspid valve as a result of entrapment of septal leaflet by device (Figure 1). Aortic cusps were intact and the valve was functioning well.

## QUESTIONS

- What is the incidence of VSD? Can all types of VSD be treated with transcatheter closure?
- What is the timing of management for children with congenital heart disease?
- What are the complications of transcatheter closure?
- What is the cause of tricuspid regurgitation?
- What are the key points to avoid tricuspid complication during a transcatheter procedure?

### Discussion of Questions

VSDs are the most common congenital heart defects affecting more than 600,000 children in the United States of America and having 2.62 prevalence per 1,000 live births worldwide^[2,3]^. In the last decades, transcatheter closure has been performed more frequently, bearing encouraging outcomes, and recently, hybrid procedures are gaining popularity^[4,5]^. These therapies challenged surgical repair since they have significant advantages like lower incidence of myocardial injury, faster recovery, shorter hospitalization, and lower cost. Thus, the number of patients undergoing surgical VSD repair decreased^[6]^. However, VSD is a more complicated pathology than secundum atrial septal defect and patent ductus arteriosus. The anatomy of the ventricular cavity challenges transcatheter procedure and patient selection. Damage to moderator band, chorda tendinea, and papillary muscle may cause significant complications. The type of VSD is important in making decisions about performing the procedure. Previously, transcatheter closure was considered to be limited to muscular VSD, which constitutes 10% of all VSD cases, meaning that an important proportion of patients with VSD and atrioventricular septal defect required open-heart surgery^[7]^. Recently, the number of reports on transcatheter closure of perimembranous VSD with good results is increasing^[8-10]^. Developments in device technology give forth to successful transcatheter closure of other types of VSD, like perimembranous defects. Amplatzer VSD occluder has been reported as safe and having good results. However, randomized, multi-center prospective further studies are needed.

Management of children with congenital heart disease depends on the severity and complexity of the disease. Infants with life-threatening condition undergo surgery as early as possible since the condition might not be compatible with life. However, in the presence of relatively less severe conditions like single septal defects, the patients are closely followed until they reach appropriate weight and growth. Sometimes, a palliative procedure is performed before total correction. In our case, device closure of the defect was performed at the right time.

The results of transcatheter procedure are encouraging, with low morbidity and mortality rates. However, it is not free of complications. Cardiac tamponade, procedure-related obstruction, and thromboembolism are life-threatening complications. Risk of peripheral vascular complications, including thrombosis, vessel injury, and arterial aneurysm, and risk of local complications, like insertion site infection and hematoma, are similar to those of any vascular intervention. Haemolysis may occur. It is usually seen soon after the procedure and disappears spontaneously in most cases. However, sometimes blood product transfusion or even surgical removal of the device may be required. Device embolization, fracture, and migration are other significant adverse events which require surgical management^[9]^. Rhythm problems are the most common complications of such procedures due to the proximity of the conductive tissue to the defect. Tachyarrhythmia, bradyarrhythmia, left bundle branch block, or right bundle branch block may emerge, and sometimes they may require medical treatment. As a more severe event, complete AV block may happen and pacemaker implantation may be needed. New-onset valve regurgitation is another significant problem. Aortic, tricuspid, or both valves may be involved. These complications occur as a result of the damage of valvar tissues like cusps, chorda tendinea, and papillary muscle. These are mostly mild regurgitation problems and disappear spontaneously over time. Surgical repair is rarely required. Although these complications are comparable to surgical treatment, it has an incidence of 12.5%. Besides, a significant complication rate is 6.5%, which should have seriously been taken into consideration^[4]^.

Tricuspid regurgitation after transcatheter VSD closure is extremely rare but it is not easy to clarify the mechanism of such complication since intra and early post-procedural follow-up may reveal only mild tricuspid regurgitation causing no symptom. There are two main types of occluders: symmetric and asymmetric. Both consist of two parts attached to each other with a micro screw mechanism. The choice of occluder mostly depends on the experience of the center and the cardiologist.

To avoid this complication, the diagnosis of such an entanglement must be made during the procedure. Mostly, the guide wire kinks while creating an arteriovenous loop. Special care should be taken not the make this kink happen. When such kink is made, it should be unwinded gently. Intraprocedural echocardiographic examination should be performed at this stage. Occluder location, residual shunt, and valve functions should be checked before leaving operating room. In-hospital and early postoperative close examinations are required and any sign of valve problem should be taken into serious consideration. The experience of the cardiologist will help with protection against such complications. Moreover, the use of new generation devices may provide more safe results. Development of right heart failure is a significant complication. Routine follow-up, early diagnosis, and accurate timing of surgery are important to provide the patient with optimum treatment.

## BRIEF CONSIDERATION OF THE CASE REPORTED

The patient was asymptomatic within the first two years after device implantation and routine follow-up showed no problem, except mild tricuspid regurgitation. Besides close follow-up, no specific management was needed. Her growth and development was within normal range. Last year before surgery, symptoms began to be prominent, slowly but progressively. At early stage of progression, a tricuspid problem was probably neglected while giving more specific care to aortic and mitral valves. When the functional capacity of the patient progressed to New York Heart Associtation Class III (parents reported that her daily activities were limited and her school success was negatively affected due to attention and concentration deficiency), ıt was seen that slow deterioration was causing heart failure. A decision of surgical management was given to prevent heart failure progression.

After interview with the cardiologist, we considered that the septal leaflet chordae got entangled within the guide wire while it was passing through the defect. However, this did not happen. The occluder was inserted properly and the guide wire was pulled back smoothly. Intra and early post-procedural examination did not reveal any significant tricuspid-related problem other than mild regurgitation. However, the stress on the septal leaflet due to the entangled chordae increased with every heartbeat. Eventually, maximum stress was reached. The chordae was not ruptured, but a cleft emerged on the free border of the related leaflet. This cleft caused a coaptation failure and finally resulted in tricuspid regurgitation.

**Fig. 2:**
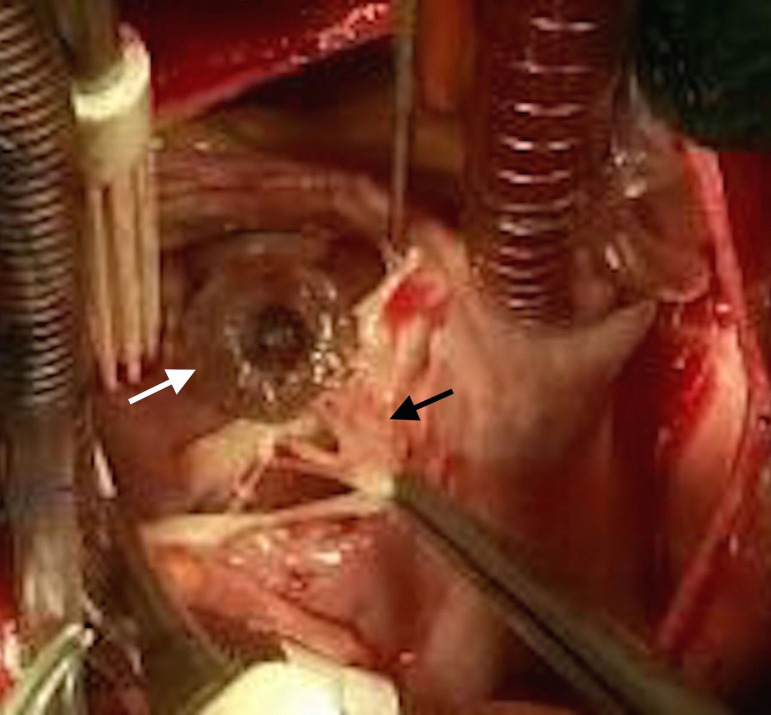
Implanted occluder device (white arrow) and septal leaflet entrapment and tear (black arrow).

Urgent surgery was performed due to the risk of developing right heart failure. After standard median sternotomy cardiopulmonary bypass was established with arterial and bicaval venous cannulation via ascending aorta and right atrium, respectively. The right atrium was opened, and the tricuspid valve was visualized. The device was intact and in the correct position. Initial inspection of the tricuspid valve revealed entrapment of septal leaflet by device causing tricuspid coaptation failure (Figure 2). After careful examination, a cleft on the free edge of the septal leaflet running through the annulus and dividing the leaflet into two parts was seen. The portion of the cusp on the occluder side was trapped by the device’s atrial disc. The opposite part was free, but unable to provide coaptation. Papillary muscle and chorda tendinea were undamaged. It was considered that septal leaflet entrapment caused a cleft within time resulting in failure of cusp coaptation, and finally, in tricuspid regurgitation.

Further examination revealed small residual VSD beneath the device, which was not clearly seen by echocardiography. The trapped part of septal leaflet was easily freed with gentle manipulations. The septal leaflet was repaired successfully and primary closure of residual VSD was performed. The device was not surgically removed since all valvar structures were undamaged, except the septal leaflet. Besides, the occluder was in close proximity of the conductive tissue and the patient was on normal sinus rhythm preoperatively. To avoid causing severe problem like damaging conductive tissue, papillary muscle, and/or chorda tendinea while extracting the device, we kept it in its implanted position and avoided over manipulation. The procedure was completed without any complications. Intraoperative transesophageal echocardiography showed competent well-functioning tricuspid valve and intact interventricular septum. No rhythm disturbance was observed. Perioperative course was uneventful. The patient did well and was discharged six days after the surgery. Echocardiographic examinations during follow-up period showed neither atrioventricular valve regurgitation nor residual interventricular shunt, but well-functioning tricuspid valve.

## LEARNING POINTS

Patients who undergo a transcatheter procedure should be monitored closely for complications.

If iatrogenic tricuspid regurgitation is diagnosed, urgent surgical treatment should be performed to prevent heart failure and related problems.

Although immediate removal of the device causing entrapment was suggested in a previous report^[10]^, the device may be left in its implanted position if there is no significant damage to tricuspid valve and if repair is possible. Over manipulation such as extraction maneuver may cause damage of structures like the conductive tissue.

**Table:** 

Authors' roles & responsibilities	
KS	Substantial contributions to the conception or design of the work; or the acquisition, analysis, or interpretation of data for the work; drafting the work or revising it critically for important intellectual content; final approval of the version to be published
AK	Substantial contributions to the conception or design of the work; final approval of the version to be published
